# Virtual Anatomy: expanding veterinary student learning

**DOI:** 10.5195/jmla.2020.1057

**Published:** 2020-10-01

**Authors:** Kyrille DeBose

**Affiliations:** 1 kdebose@vt.edu, Associate Professor, Veterinary Medicine Library, Virginia Tech, Blacksburg, VA

## Abstract

Traditionally, there are three primary ways to learn anatomy outside the classroom. Books provide foundational knowledge but are limited in terms of object manipulation for deeper exploration. Three-dimensional (3D) software programs produced by companies including Biosphera, Sciencein3D, and Anatomage allow deeper exploration but are often costly, offered through restrictive licenses, or require expensive hardware. A new approach to teaching anatomy is to utilize virtual reality (VR) environments. The Virginia–Maryland College of Veterinary Medicine and University Libraries have partnered to create open education–licensed VR anatomical programs for students to freely download, access, and use. The first and most developed program is the canine model. After beta testing, this program was integrated into the first-year students' physical examination labs in fall 2019. The VR program enabled students to walk through the VR dog model to build their conceptual knowledge of the location of certain anatomical features and then apply that knowledge to live animals. This article briefly discusses the history, pedagogical goals, system requirements, and future plans of the VR program to further enrich student learning experiences.

Traditional methods of teaching anatomy in a veterinary curriculum include using textbooks, live animals, and cadavers to build foundational knowledge. Three-dimensional (3D) software programs provide a platform for deeper examination of anatomical structures but are unable to deliver a fully immersive experience. The use of virtual reality (VR) provides a new type of learning environment.

After a comprehensive curriculum change occurred in 2016 at the Virginia–Maryland College of Veterinary Medicine (VMCVM), faculty discovered students no longer possessed a conceptual picture of anatomy during lab sessions. The idea to use VR to bridge this knowledge gap was developed through a collaboration among faculty members in the VMCVM, School of Visual Arts, and University Libraries. The library provided a $3,000 Open Educational Resource (OER) grant to launch the project.

The project was developed using Unreal Engine 4 because it supported the ability to manipulate and control the displayed levels of anatomy. Data derived from CT scans was sliced using Slic3r to create high-quality topology models for each anatomical structure. Models were exported into other 3D modeling programs for UV unwrapping, projection of a two-dimensional surface to a 3D model, to provide texture applications and re-topologize the graphics for better integration and rendering in Unreal Engine. Users were able to access the program in the VR environment through SteamVR.

Because first-year students used live canines to develop their clinical skills, the dog was the first VR model to be developed. After beta testing, this program was integrated into the first-year students' physical examination labs in fall 2019. Although still under development, the VR program enabled students to walk through the VR dog model to build their conceptual knowledge of anatomical features and relationships. Students then applied that knowledge as they determined physiological markers from the program to use when palpating and examining the dogs.

Students voluntarily shared their experiences and perceptions using VR as part of their learning environment. Prior to using VR in the first lab, 20% of respondents stated they had previous experience using a VR system and 26% were moderately familiar with anatomy. After the first lab, 40% indicated the VR program was very or extremely effective for learning anatomical features, 78% indicated the labeling was effective, and 83% stated they could transfer the anatomical locations from the VR dog model to the live dog. After the last lab, 57% of respondents stated a high or very high likelihood of continuing to use VR as a learning tool.

Additional funding from other sources has been procured, and the library's role has evolved into a full partnership to continue advancing the VR dog model and to develop VR cow and horse models. Currently, the program works with Oculus Rift CV1 and HTC Vive VR headset and controllers, but developments are being made for additional wireless options like Oculus Quest. Because the equipment can be cost-prohibitive, the Veterinary Medicine Library provides training and access for students in the library. Another group is examining how the program will work in less expensive VR systems and eventually on smartphones. Additionally, work is being done to incorporate haptic technologies for the cow and horse models to further enhance the learning opportunities available through VR.

It is also important to note two philosophical goals that were implemented at the beginning of the project. The first is that all anatomical programs that are developed will be freely available for anyone to download, access, and use through an OER license. The second is to develop a community of users and contributors who can add files that will enable new models to be created, such as models that include common abnormalities; to support additional learning objectives at a significantly reduced cost; and to allow the new model to be shared with others. For more information and a video on how VR is currently being used by the first-year class, visit Vet Med Library Information: Virtual Reality Anatomical Models.

